# Effectiveness of Standard-Dose vs. Low-Dose Alteplase for Acute Ischemic Stroke Within 3–4.5 h

**DOI:** 10.3389/fneur.2022.763963

**Published:** 2022-02-08

**Authors:** Chih-Hao Chen, Sung-Chun Tang, Yu-Wei Chen, Chih-Hung Chen, Li-Kai Tsai, Sheng-Feng Sung, Huey-Juan Lin, Hung-Yu Huang, Helen L. Po, Yu Sun, Po-Lin Chen, Lung Chan, Cheng-Yu Wei, Jiunn-Tay Lee, Cheng-Yang Hsieh, Yung-Yang Lin, Li-Ming Lien, Jiann-Shing Jeng

**Affiliations:** ^1^Stroke Center and Department of Neurology, National Taiwan University Hospital, Taipei, Taiwan; ^2^Department of Neurology, Landseed International Hospital, Taoyuan, Taiwan; ^3^Department of Neurology, National Cheng Kung University Hospital, Tainan, Taiwan; ^4^Division of Neurology, Department of Internal Medicine, Ditmanson Medical Foundation Chiayi Christian Hospital, Chiayi, Taiwan; ^5^Department of Neurology, Chi Mei Medical Center, Tainan, Taiwan; ^6^Department of Neurology, China Medical University Hospital, Taichung, Taiwan; ^7^Department of Neurology, Mackay Memorial Hospital, Taipei, Taiwan; ^8^Department of Neurology, En Chu Kong Hospital, New Taipei City, Taiwan; ^9^Department of Neurology, Taichung Veterans General Hospital, Taichung, Taiwan; ^10^Department of Neurology and Stroke Center, Taipei Medical University–Shuang Ho Hospital, New Taipei City, Taiwan; ^11^Department of Neurology, Chang Bing Show Chwan Memorial Hospital, Changhwa, Taiwan; ^12^Department of Neurology, Tri Service General Hospital, Taipei, Taiwan; ^13^Department of Neurology, Tainan Sin-Lau Hospital, Tainan, Taiwan; ^14^Department of Neurology and Department of Critical Care Medicine, Taipei Veterans General Hospital, Taipei, Taiwan; ^15^Department of Neurology, Shin Kong WHS Memorial Hospital, Taipei, Taiwan

**Keywords:** thrombolysis, alteplase, rt-PA, atrial fibrillation, hypercholesterolemia

## Abstract

**Background:**

The efficacy and safety of intravenous alteplase administered 3–4.5 h after acute ischemic stroke have been demonstrated. However, whether responses differ between low-dose and standard-dose alteplase during this time window and whether certain subgroups benefit more remain unknown.

**Patients and Methods:**

The current analysis was based on a multicenter matched-cohort study conducted in Taiwan. The treatment group comprised 378 patients receiving intravenous alteplase 3–4.5 h after stroke onset, and the control group comprised 378 age- and sex-matched patients who did not receive alteplase treatment during the same period. Standard- and low-dose alteplase was administered to patients at the physician's discretion.

**Results:**

Overall, patients receiving alteplase exhibited more favorable outcomes than did controls [34.0 vs. 22.7%; odds ratio (OR): 1.75, 95% confidence interval (CI): 1.27–1.42], and the effectiveness was consistent in all subgroups. Although patients in the standard-dose group (*n* = 182) were younger than those in the low-dose (*n* = 192) group, the proportions of patients with favorable outcomes (36.3 vs. 31.8%; OR: 1.22, 95% CI: 0.80–1.88) and symptomatic hemorrhage (2.8 vs 4.2%; OR: 0.65, 95% CI: 0.21–2.02) were consistently comparable in a covariate-adjusted model and an age-matched cohort. In the subgroup analysis, patients with cardioembolism, atrial fibrillation, and hypercholesterolemia were more likely to achieve favorable outcomes after receiving standard-dose than low-dose alteplase.

**Conclusion:**

In the 3–4.5 h time window, the effectiveness and safety of standard-dose and low-dose alteplase may be comparable. A standard dose may be selected for patients with cardioembolism, atrial fibrillation, or hypercholesterolemia.

## Introduction

The efficacy and safety of intravenous thrombolysis with 0.9 mg/kg alteplase at 3–4.5 h after acute ischemic stroke (AIS) were first demonstrated in the European Cooperative Acute Stroke Study III [ECASS III; (1)] and have been subsequently verified by a meta-analysis (2) and several real-world studies (3–5). Intravenous alteplase treatment for AIS within 4.5 h of symptom onset is currently recommended by various professional organizations (6–9). However, the Food and Drug Administrations in the United States and Taiwan have yet to approve the use of alteplase in the time window of 3–4.5 h.

Whether a low dose of alteplase reduces the risk of intracerebral hemorrhage (ICH) with similar effectiveness as that of a standard dose has long been debated. The ENhanced Control of Hypertension And Thrombolysis strokE stuDy (ENCHANTED) demonstrated that although low-dose alteplase was not non-inferior to standard-dose alteplase in reducing death and disability when used within 4.5 h of stroke onset, significantly fewer symptomatic ICH (sICH) events were reported in the low-dose group than in the standard-dose group (10). Currently, 0.6 mg/kg is the only approved low dose for alteplase in Japan; moreover, low-dose alteplase is commonly used in several other Asian countries, including Taiwan, for safety and cost reduction (11).

Most studies comparing standard-dose and low-dose alteplase have included patients treated within 3 or 4.5 h; however, few studies have specifically emphasized the time window of 3–4.5 h. Because the response to alteplase treatment may gradually decrease with time, whether a low dose can achieve similar effectiveness as that of a standard dose in the time window of 3–4.5 h remains unknown. Additionally, whether certain patient subgroups may benefit more from standard-dose or low-dose alteplase merits investigation. Therefore, in this study, we analyzed data of a published multicenter matched-cohort study in Taiwan that had demonstrated the real-world effectiveness of alteplase administered in the time window of 3–4.5 h after symptom onset (12).

## Methods

### Study Design

This study involved the analysis of data from a multicenter, retrospective, matched-cohort study initiated by the Taiwan Stroke Society to evaluate the effectiveness and safety of intravenous alteplase at 3–4.5 h after symptom onset in patients with AIS. The detailed study protocol and results have been published, and the present study is a subgroup analysis of the primary study (12). Briefly, data were extracted from 16 hospitals participating in the Taiwan Stroke Registry, which contains prospectively collected data on patients' basic demographic characteristics and risk factors, clinical course and treatment, and etiology and outcomes of stroke. The study period was from January 2008 to December 2017. The use of data from the Taiwan Stroke Registry was approved by the Institutional Review Board of National Taiwan University Hospital (Research Ethical Committee No. 201801064RINC) and informed consent was waived because this was a retrospective analysis of the prospective stroke registry. All study methods were performed in accordance with the Declaration of Helsinki. The data used in the present study can be obtained from the corresponding author on reasonable request.

### Study Population

Although this was not a randomized controlled trial, we enrolled patients at a 1:1 ratio according to whether they received intravenous alteplase (treatment group) or not (control group) within the specified time window. Patients ≥18 years old with a clinical diagnosis of AIS were included in the analysis. Patients in the treatment group received intravenous alteplase within a time window of 3–4.5 h after stroke onset; their treatment complied with the regulations of the Taiwan Food and Drug Administration and the reimbursement criteria of the National Health Insurance program in Taiwan (13). For each patient in the treatment group, one age- and sex-matched patient arriving at the emergency room in the same hospital within 2–4.5 h, but not receiving intravenous alteplase, was enrolled into the control group. The rationale behind selecting 2 h as a lower limit of onset-to-door time was that thrombolysis could not be administered to these patients within 3 h. In addition, patients were assigned to the control group only if they did not have any obvious contraindication to intravenous alteplase. Patients who received any other reperfusion therapy such as intra-arterial thrombolysis or endovascular thrombectomy were excluded from the current analysis.

The Taiwan Food and Drug Administration has approved the administration of 0.9 mg/kg intravenous alteplase for AIS as the standard dose in clinical practice. In addition, the Taiwan Food and Drug Administration has recommended that low-dose (0.6 mg/kg) intravenous alteplase may be associated with lower sICH risk on the basis of the results in the ENCHANTED and Taiwan Thrombolytic Therapy for Acute Ischemic Stroke (TTT-AIS) trials (10, 14). In clinical practice, a standard or low dose is used according to the treating physicians' initial evaluation of the patient and professional discretion. Generally, physicians in Taiwan prefer using low-dose alteplase in patients >70 years old on the basis of the results of the TTT-AIS study (14).

### Clinical Characteristics and Outcome Measures

The demographic profile, body weight, and vascular risk factors (namely hypertension, diabetes mellitus, hyperlipidemia, previous ischemic stroke, ischemic heart disease, atrial fibrillation, and ever or current smoking) of the study patients were documented. Furthermore, the initial National Institute of Health Stroke Scale (NIHSS) score, blood pressure, and laboratory data (including glucose, creatinine, platelets, and international normalized ratio) at the index stroke event were recorded. The NIHSS score was recorded at least once every day in the first 3 days and then recorded according to regional clinical practice. Hyperlipidemia was defined as receiving of lipid-lowering agents or having one of the following: fasting serum total cholesterol ≥ 200 mg/dl, fasting serum low-density lipoprotein cholesterol ≥ 130 mg/dl, fasting serum high-density lipoprotein <40 mg/dl, or fasting serum triglyceride ≥ 150 mg/dl. Hyperlipidemia was further classified as hypercholesterolemia (total cholesterol ≥ 200 mg/dl or low-density lipoprotein cholesterol ≥ 130 mg/dl) or hypertriglyceridemia (triglyceride ≥ 150 mg/dl). Ischemic stroke was classified into the subtypes large-artery atherosclerosis, small vessel occlusion, cardioembolism, and others on the basis of the Trial of Org 10172 in the Acute Stroke Treatment (TOAST) classification (15).

The primary effectiveness outcome was the percentage of patients with favorable functional outcomes as defined by modified Rankin Scale (mRS) scores of 01 at 90 days after index stroke event. The secondary effectiveness outcomes were the percentage of patients with mRS scores of 02 at 90 days and early neurological deterioration (END), which was defined as an increase in the NIHSS score by two or more points from the initial or the lowest NIHSS score between the time of admission and 72 h. The safety outcomes were any ICH event after thrombolysis and sICH occurrence as defined by the ECASS III criteria (1). To detect ICH, a brain computed tomography or magnetic resonance imaging scan was routinely performed at 24–36 h after thrombolysis.

### Statistical Analysis

The original study consisted of 748 eligible patients (original cohort), of whom 374 received alteplase (alteplase cohort) and 374 did not receive alteplase (control cohort). In the original cohort, the baseline characteristics were comparable, as reported in the published article [Supplemental Table 1; (12)]. The demographic, clinical, and laboratory profiles were compared between the standard-dose and low-dose groups in the alteplase cohort by using the Mann–Whitney *U* test or chi-square test, as appropriate. An additional comparison was performed between patients treated with alteplase (standard and low doses) and controls. Because a considerable age gap existed between patients treated with standard and low doses of alteplase, the cohorts were age- and NIHSS score–matched by using the SAS PSMATCH procedure.

The effectiveness and safety outcomes (expressed in percentage) of the standard-dose and low-dose groups in the alteplase cohort were plotted by age at 10-year intervals (<50, 5059, 6069, 7079, and ≥80), and the trend between the age groups and alteplase dose was tested using the generalized linear mixed model. To compare clinical outcomes between the standard-dose and low-dose groups, logistic regression analyses were performed with effectiveness (mRS scores 01, mRS scores 02, and END) and safety outcomes (any ICH and sICH event) as dependent variables. First, an unadjusted analysis was performed, and the crude odds ratio (OR) was calculated. Subsequently, a multivariable analysis was performed and adjusted for covariates that were significantly associated with outcomes in the univariate analysis (Supplemental Table 2). The covariates were age, NIHSS score, diabetes mellitus, previous ischemic stroke, and atrial fibrillation for mRS score 01; NIHSS score, diabetes mellitus, and atrial fibrillation for END; and male sex, NIHSS score, diabetes mellitus, and atrial fibrillation for any ICH event. Symptomatic ICH was not adjusted for in the analysis owing to its rarity. Furthermore, unadjusted logistic regression analyses were performed between the matched patients of the standard-dose and low-dose groups.

To explore which subgroup may benefit more from the treatment (alteplase vs. control or standard dose vs. low dose), logistic regression analyses were performed with effectiveness outcomes as the dependent variable and clinical variables, treatment, and interaction terms between the clinical variables and treatment as the predictors. Statistically significant interaction terms in the subgroup analyses implied that treatment effects may differ in the subgroup. All statistical analyses were performed using SAS version 9.4 (SAS Institute Inc, Cary, NC, USA), and a *P*-value of < 0.05 indicated significance.

## Results

### Patient Characteristics

Baseline demographics, vascular risk factors, laboratory results, and stroke profiles of patients in the standard-dose alteplase (*n* = 182), low-dose alteplase (*n* = 192), and control (*n* = 374) groups are summarized in Table 1. Patients in the low-dose group were significantly older than those in the standard-dose group (69.5 ± 12.4 vs. 62.5 ± 13.1 years, *P* < 0.0001). The door-to-needle time was comparable between the two groups, whereas the onset-to-needle time was longer in the low-dose group. The age- and NIHSS score–matched cohorts comprised 65 patients each at a 1:1 ratio. The median age was 68 years in both the groups, and their demographic profiles were comparable (Supplemental Table 3).

**Table 1:** Baseline characteristics of patients who were given standard-dose and low-dose alteplase vs. controls.

**Characteristics**	**Standard dose**	**Low dose**	**Control**
	**(*n* = 182)**	**(*n* = 192)**	**(*n* = 374)**
Age (year)	63 (53–72)	72 (63–79)^*^	69 (60–77)^†^
Age≥70 y, *n* (%)	53 (29.1)	107 (55.7)^*^	183 (48.9)^†^
Age≥80 y, *n* (%)	16 (8.8)	40 (20.8)	70 (18.7)^†^
Male sex, *n* (%)	123 (67.6)	128 (66.7)	251 (67.1)
Body weight (Kg)	63 (56–75)	65 (56–71)	65 (56–73)
**Stroke subtype**			
LAA	40 (22.0)	47 (24.5)	97 (25.9)
SVO	32 (17.6)	22 (11.5)	75 (20.1)
CE	52 (28.6)	66 (34.4)	108 (28.9)
Others	58 (31.9)	57 (29.7)	94 (25.1)
NIHSS	11 (7–17)	10 (6–17)	9 (5–15)^†^^,^^‡^
Systolic BP (mmHg)	156 (136–181)	158 (141–182)	161 (138–182)
Diastolic BP (mmHg)	92 (81–106)	88 (77–100)^*^	90 (79–102)
Onset-to-needle (min)	195 (184–215)	205 (190–238)^*^	-
Door-to-needle (min)	67 (51–99)	64 (50–87)	-
**Medical history**			
Hypertension	136 (74.7)	146 (76.0)	291 (77.8)
Diabetes mellitus	62 (34.1)	75 (39.1)	160 (42.8)^†^
Previous stroke	32 (17.6)	43 (22.4)	97 (25.9)^†^
Diabetes mellitus with previous stroke	14 (7.7)	23 (12.0)	45 (12.0)
Ischemic heart disease	19 (10.4)	26 (13.5)	38 (10.2)
Atrial fibrillation	55 (30.2)	72 (37.5)	113 (30.2)
Hyperlipidemia	101 (55.5)	98 (51.0)	201 (53.7)
Hypercholesterolemia	82 (45.1)	92 (47.9)	175 (46.8)
Hypertriglyceridemia	40 (22.0)	27 (14.1)^*^	64 (17.1)
Ever smoking	57 (31.3)	71 (37.0)	137 (36.6)
Current smoker	51 (28.0)	52 (27.1)	98 (26.2)
Prior antiplatelet use	35 (19.2)	47 (24.5)	99 (26.5)
Prior anticoagulant use	5 (2.8)	5 (2.6)	16 (4.3)
**Laboratory data**			
Glucose (mg/dl)	132 (112–172)	133 (105–180)	130 (110–182)
INR	1.00 (0.96–1.07)	0.99 (0.94–1.05)	1.00 (0.95–1.06)
Creatinine (mg/dl)	1.01 (0.85–1.34)	1.00 (0.80–1.27)	1.00 (0.80–1.30)
Platelet count (10^5^/mm^3^)	221 (178–258)	201 (166–236)^*^	203 (168–251)^†^
**Outcome**			
mRS 0–1	66 (36.3)	61 (31.8)	85 (22.7)^†^^,^^‡^
mRS 0–2	92 (50.6)	87 (45.3)	150 (40.1)^†^
END	23 (12.6)	33 (17.2)	73 (19.5)^†^
Any ICH	29 (15.9)	36 (18.8)	32 (8.6)^†^^,^^‡^
Symptomatic ICH	5 (2.8)	8 (4.2)	9 (2.4)

*All variables were compared between patients receiving standard-dose and the low-dose alteplase first. Additional comparisons were performed between the controls and standard-dose or low-groups*.

* *P < 0.05 between the standard-dose and the low-dose groups*.

† *P < 0.05 between the standard-dose group and control*;

‡ *P < 0.05 between the low-dose group and control*.

*BP, blood pressure; CE, cardioembolism; END, early neurological deterioration; ICH, intracerebral hemorrhage; INR, international normalized ratio; mRS, modified Rankin Scale; LAA, large-artery atherosclerosis; NIHSS, national institute of health stroke scale; SVO, small vessel occlusion*.

### Clinical Outcomes Between Patients Treated With Standard-Dose and Low-Dose Alteplase

**Figure 1:**
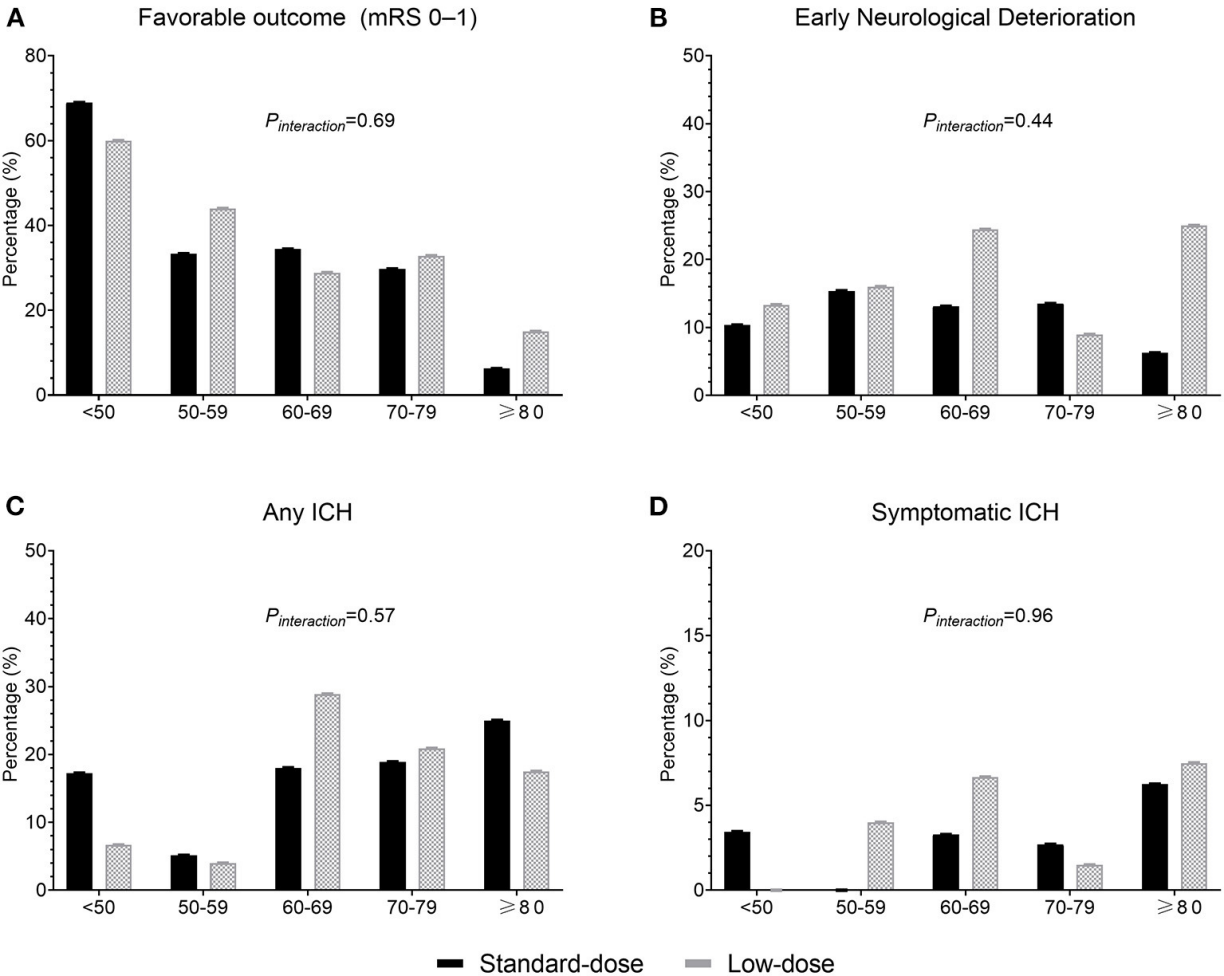
Proportions of clinical outcomes between the standard-dose and low-dose groups according to age. **(A)** Favorable outcome (modified Rankin Scale score 01); **(B)** early neurological deterioration; **(C)** any intracerebral hemorrhage (ICH); and **(D)** symptomatic ICH.

Figure 1 presents the distribution of favorable functional outcomes (mRS scores 01), END, any ICH event, and sICH by age at the 10-year intervals between the standard-dose and low-dose groups. Despite the trend of fewer favorable outcomes (*P*_*trend*_ = 0.0002) and more ICH events (*P*_*trend*_ = 0.06) with increasing age, no significant age and dose interaction effect was observed for all clinical outcomes. For mRS scores 0–1, 36.3% and 31.8% of patients exhibited the primary effectiveness outcome in the standard-dose and low-dose groups, respectively (OR = 1.22, 95% confidence interval [CI] = 0.80–1.88, *P* = 0.36). For mRS scores 02, the corresponding proportions were 50.6% and 45.3% (OR = 1.23, 95% CI = 0.82–1.85). Compared with the low-dose group, fewer patients reported END, any ICH event, and sICH in the standard-dose group, although the results were nonsignificant (Table 2).

**Table 2:** Outcomes of patients receiving standard-dose and low-dose alteplase.

**Clinical outcome**	**Standard-dose**	**Low-dose**	**Crude OR (95% CI)**	**Covariate-adjusted OR (95% CI)**	**Matched cohort OR (95% CI)**
mRS 0–1	66 (36.26)	61 (31.77)	1.22 (0.80–1.88)	0.96 (0.85–1.61)	0.87 (0.43–1.80)
mRS 0–2	92 (50.55)	87 (45.31)	1.23 (0.82–1.85)	1.08 (0.65–1.78)	0.94 (0.47–1.87)
END	23 (12.64)	33 (17.19)	0.70 (0.39–1.24)	0.75 (0.41–1.37)	0.89 (0.35–2.27)
Any ICH	29 (15.93)	36 (18.75)	0.82 (0.48–1.41)	0.86 (0.47–1.56)	0.88 (0.33–2.34)
Symptomatic ICH	5 (2.75)	8 (4.17)	0.65 (0.21–2.02)	0.65 (0.21–2.02)^*^	0.49 (0.04–5.57)

* *Symptomatic ICH outcome was not further adjusted with covariates owing to its rarity*.

*END, early neurological deterioration; ICH, intracerebral hemorrhage; mRS, modified Rankin Scale*.

**Figure 2:**
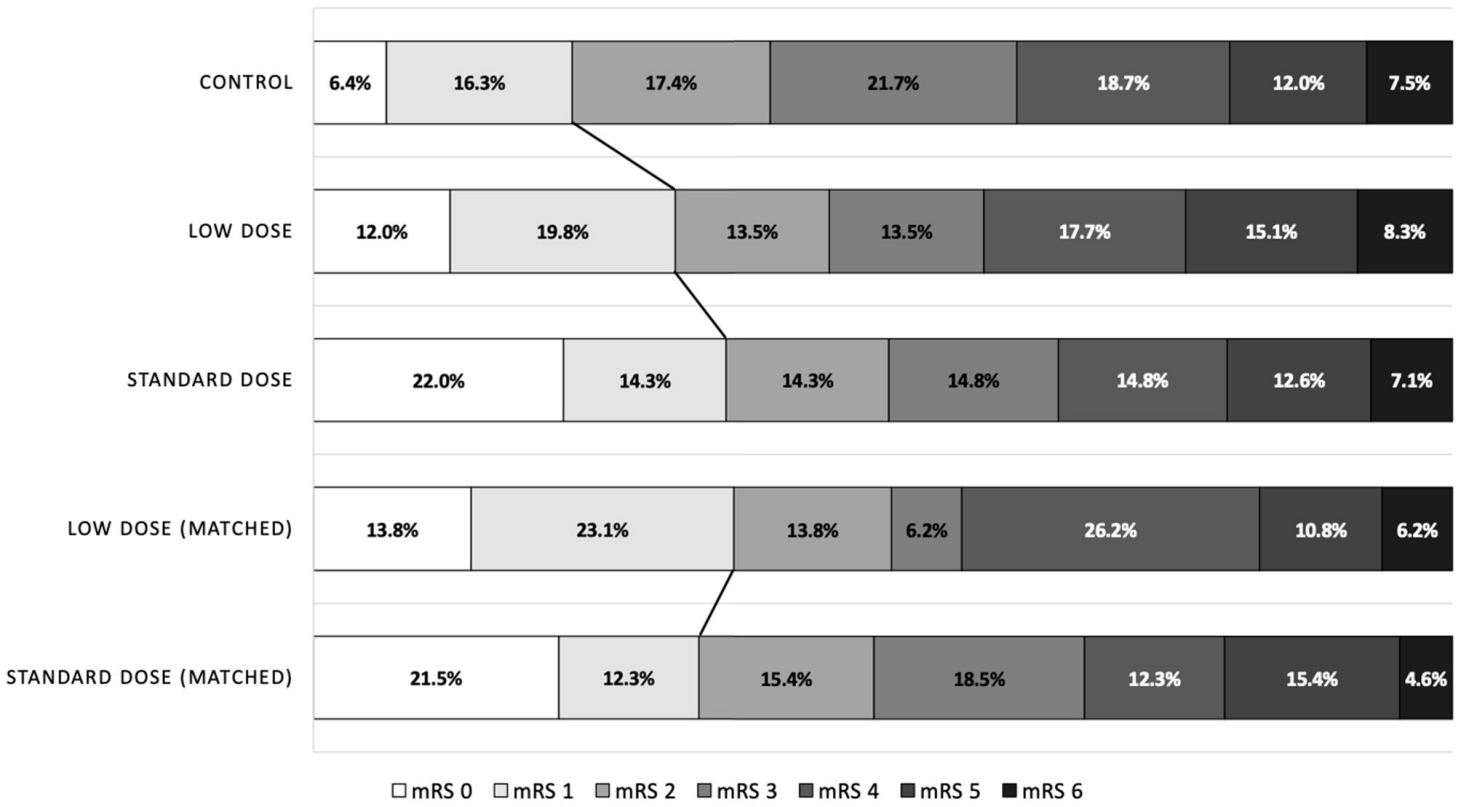
Distribution of modified Rankin Scale scores at 90 days after stroke, according to the treatment groups.

In the covariate-adjusted model, the results were overall comparable when the point estimates moved toward null as expected. However, in the matched cohort, the proportions of patients exhibiting favorable outcome were 33.9% and 36.9% in the standard-dose and low-dose groups, respectively (OR = 0.87, 95% CI = 0.43–1.80; Figure 2). The point estimates of other outcomes were similar to those of the alteplase cohort.

### Subgroup Analysis of Effectiveness Outcomes

**Figure 3:**
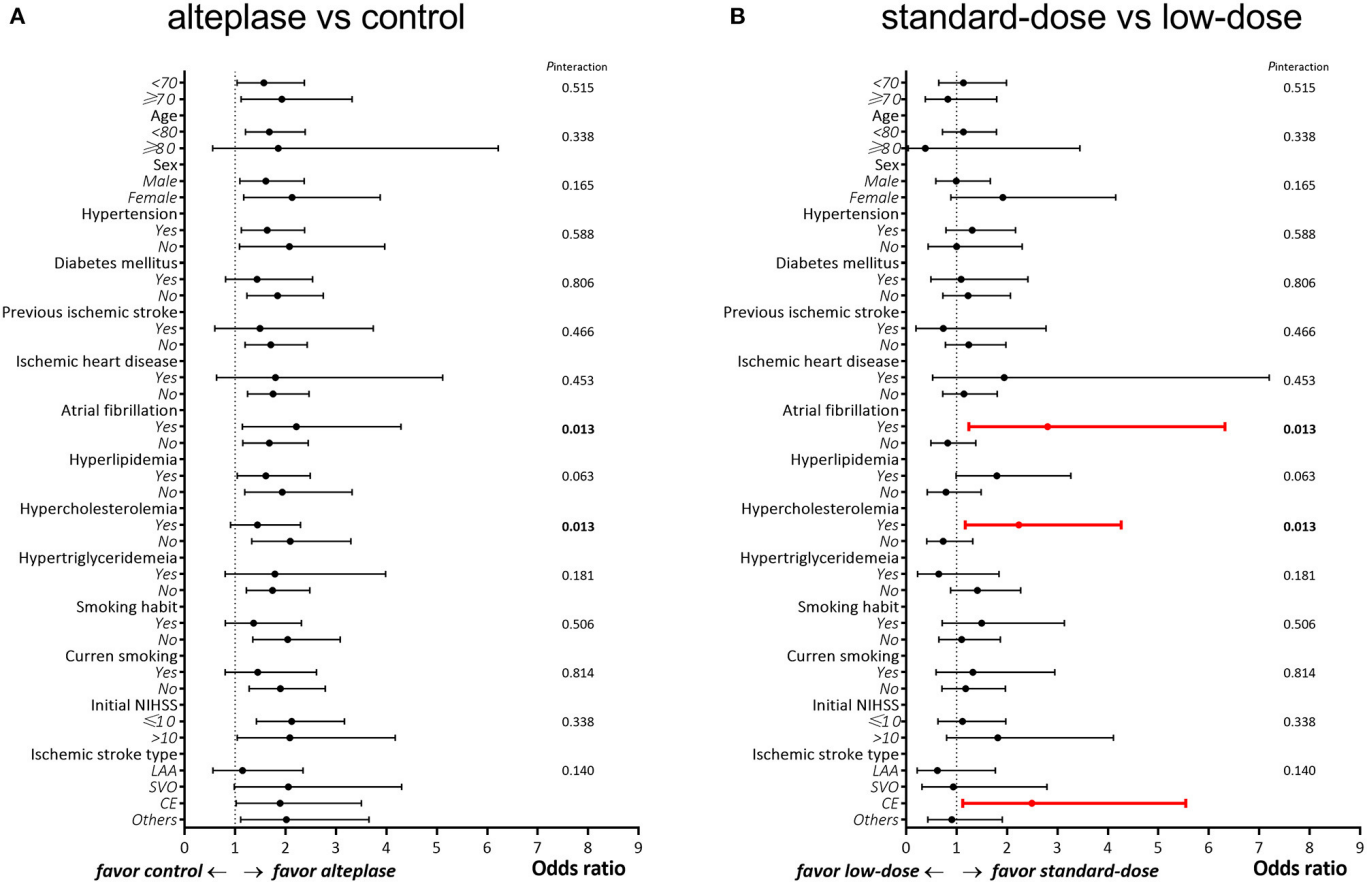
Subgroup analysis between patients treated with **(A)** alteplase vs. control and **(B)** standard-dose vs. low-dose alteplase.

In the original cohort (alteplase vs. control), no clinical variable significantly influenced the primary outcome (all *P*_*interaction*_ > 0.05; Table 3 and Figure 3A), indicating that the effectiveness of alteplase was consistent in all patient subgroups. These included subgroups that would otherwise be excluded from the ECASS III trial, such as patients aged >80 years (*P*_*interaction*_ = 0.89), those with concomitant diabetes mellitus and prior stroke (*P*_*interaction*_ = 0.37), and those using oral anticoagulants (*P*_*interaction*_ = 0.15).

**Table 3:** Subgroup analysis of the effectiveness of alteplase in yielding favorable outcome.

**Variables**		***N***	**OR**	**95% CI**	**P_**interaction**_**
Age	<70 y	405	1.571	1.040–2.373	0.558
	≥70 y	343	1.926	1.118–3.318	
	<80 y	622	1.697	1.205–2.389	0.888
	≥80 y	116	1.857	0.555–6.216	
Sex	Male	502	1.611	1.095–2.370	0.441
	Female	246	2.131	1.172–3.877	
Hypertension	Yes	573	1.636	1.124–2.379	0.530
	No	175	2.078	1.088–3.967	
Diabetes mellitus	Yes	297	1.437	0.813–2.538	0.483
	No	451	1.843	1.235–2.749	
Previous stroke	Yes	172	1.495	0.598–3.740	0.791
	No	576	1.707	1.199–2.430	
Diabetes mellitus with previous stroke	Yes	82	3.267	0.779–13.701	0.370
	No	666	1.666	1.191–2.329	
Ischemic heart disease	Yes	83	1.799	0.633–5.115	0.964
	No	665	1.754	1.247–2.466	
Atrial fibrillation	Yes	240	2.216	1.146–4.288	0.475
	No	508	1.680	1.152–2.451	
Hyperlipidemia	Yes	400	1.610	1.041–2.490	0.579
	No	348	1.936	1.194–3.139	
Hypercholesterolemia	Yes	349	1.444	0.909–2.296	0.262
	No	399	2.094	1.330–3.296	
Hypertriglyceridemia	Yes	131	1.444	0.909–2.296	0.952
	No	617	2.094	1.330–3.296	
Smoking habit	Yes	255	1.367	0.807–2.316	0.240
	No	483	2.042	1.351–3.085	
Current smoking	Yes	201	1.450	0.804–2.615	0.462
	No	547	1.889	1.281–2.787	
Prior antiplatelet use	Yes	181	1.111	0.542–2.278	0.181
	No	567	1.924	1.334–2.775	
Prior anticoagulant use	Yes	26	10.00	0.915–109.2	0.145
	No	722	1.665	1.200–2.310	
Initial NIHSS score	≤10	202	2.122	1.421–3.169	0.966
	>10	546	2.086	1.042–4.174	
Ischemic stroke types	LAA	184	1.147	0.561–2.346	0.614
	SVO	129	2.057	0.984–4.031	
	CE	226	1.892	1.022–3.504	
	Others	209	2.016	1.112–3.656	

*Favorable outcome was defined as a modified Rankin Scale score of 0 or 1*.

*CE, cardioembolism; LAA, large-artery atherosclerosis; NIHSS, national institute of health stroke scale; SVO, small vessel occlusion*.

In the alteplase cohort (standard dose vs. low dose), significant interactions were observed between the alteplase dose and the presence of atrial fibrillation (*P*_*interaction*_ = 0.01) as well as between the alteplase dose and the presence of hypercholesterolemia (*P*_*interaction*_ = 0.01; Table 4 and Figure 3B). In patients with atrial fibrillation, administration of a standard dose resulted in higher odds of favorable outcomes than using a low dose (OR = 2.80, 95% CI = 1.24–6.32); in patients without atrial fibrillation, opposite results were obtained (OR = 0.82, 95% CI = 0.49–1.38). Furthermore, administration of a standard dose was associated with favorable outcome in patients with hypercholesterolemia (OR = 2.23, 95% CI = 1.17–4.26) but not in patients without hypercholesterolemia (OR = 0.73, 95% CI = 0.41–1.32). Regarding ischemic stroke type, administration of standard-dose alteplase was associated with favorable outcome only in patients with the cardioembolism subtype (OR = 2.49, 95% CI = 1.12–5.54). The interaction effect between the alteplase dose and atrial fibrillation remained significant even after adjustment for age and NIHSS score (*P*_*interaction*_ = 0.03) or in the matched cohort (*P*_*interaction*_ = 0.04). On the other hand, the interaction effect between the dose and hypercholesterolemia persisted in the age- and NIHSS score–adjusted model (*P*_*interaction*_ = 0.04) but not in the matched cohort (*P*_*interaction*_ = 0.38). No significant interaction was noted between the occurrence of any ICH event and clinical variables (all *P*_*interaction*_ > 0.05; Table 5).

**Table 4:** Subgroup analysis of the effectiveness of standard-dose vs. low-dose alteplase.

**Variables**		***N***	**OR**	**95% CI**	***P_**interaction**_***
Age strata	<70 y	214	1.14	0.65–1.99	0.52
	≥70 y	160	0.83	0.38–1.80	
	<80 y	318	1.14	0.72–1.79	0.34
	≥80 y	56	0.38	0.04–3.44	
Sex	Male	251	0.99	0.59–1.67	0.17
	Female	123	1.92	0.89–4.15	
Hypertension	Yes	282	1.31	0.79–2.17	0.59
	No	92	1.00	0.44–2.30	
Diabetes mellitus	Yes	137	1.09	0.49–2.41	0.81
	No	237	1.23	0.73–2.07	
Previous stroke	Yes	75	0.74	0.16–2.77	0.47
	No	299	1.24	0.78–1.98	
Diabetes mellitus with previous stroke	Yes	37	0.22	0.02–2.06	0.12
	No	337	1.31	0.84–2.05	
Ischemic heart disease	Yes	45	1.94	0.53–7.20	0.45
	No	329	1.15	0.73–1.81	
Atrial fibrillation	Yes	127	**2.80**	**1.24–6.32**	**0.01**
	No	247	0.82	0.49–1.38	
Hyperlipidemia	Yes	199	1.80	0.99–3.26	0.06
	No	175	0.79	0.42–1.49	
Hypercholesterolemia	Yes	174	**2.23**	**1.17–4.26**	**0.01**
	No	200	0.73	0.41–1.32	
Hypertriglyceridemia	Yes	67	0.65	0.23–1.84	0.18
	No	307	1.41	0.88–2.27	
Smoking habit	Yes	128	1.50	0.71–3.14	0.51
	No	246	1.10	0.65–1.87	
Current smoking	Yes	103	1.32	0.59–2.95	0.81
	No	271	1.18	0.71–1.97	
Prior antiplatelet use	Yes	82	1.10	0.38–3.16	0.88
	No	292	1.20	0.75–1.93	
Prior anticoagulant use	Yes	10	1.00	0.08–12.7	0.88
	No	364	1.23	0.79–1.90	
Initial NIHSS score	≥10	191	1.12	0.63–1.97	0.34
	>10	183	1.82	0.80–4.11	
Ischemic stroke types	LAA	87	0.62	0.22–1.77	0.14
	SVO	54	0.93	0.31–2.79	
	CE	118	**2.49**	**1.12–5.54**	
	Others	115	0.90	0.43–1.91	

*Favorable outcome was defined as a modified Rankin Scale score of 0 or 1*.

*CE, cardioembolism; LAA, large-artery atherosclerosis; NIHSS, national institute of health stroke scale; SVO, small vessel occlusion*.

*Values in bold indicate statistical significance*.

**Table 5:** Subgroup analysis of the safety (all ICH) of standard-dose vs. low-dose alteplase.

**Variables**		***N***	**OR**	**95% CI**	**P_**interaction**_**
Age	<70 y	214	0.757	0.357–1.602	0.538
	≥70 y	160	1.073	0.472–2.436	
	<80 y	318	0.752	0.417–1.355	0.341
	≥80 y	56	1.571	0.388–6.369	
Sex	Male	251	0.914	0.442–1.890	0.674
	Female	123	0.722	0.317–1.648	
Hypertension	Yes	282	0.745	0.406–1.373	0.485
	No	92	1.197	0.368–3.895	
Diabetes mellitus	Yes	137	0.941	0.430–2.058	0.731
	No	237	0.777	0.363–1.663	
Previous stroke	Yes	75	1.010	0.311–3.278	0.706
	No	299	0.783	0.426–1.436	
Diabetes mellitus with previous stroke	Yes	37	1.133	0.255–5.037	0.680
	No	337	0.810	0.453–1.449	
Ischemic heart disease	Yes	45	2.538	0.599–10.754	0.100
	No	329	0.688	0.382–1.238	
Atrial fibrillation	Yes	127	0.582	0.264–1.284	0.121
	No	247	1.428	0.633–3.225	
Hyperlipidemia	Yes	199	0.657	0.302–1.429	0.405
	No	175	1.039	0.490–2.207	
Hypercholesterolemia	Yes	174	0.507	0.213–1.205	0.156
	No	200	1.139	0.560–2.316	
Hypertriglyceridemia	Yes	67	0.629	0.162–2.434	0.655
	No	307	0.879	0.488–1.585	
Smoking habit	Yes	128	0.524	0.170–1.614	0.398
	No	246	0.912	0.487–1.710	
Current smoking	Yes	103	0.407	0.116–1.423	0.211
	No	271	0.987	0.538–1.811	
Prior antiplatelet use	Yes	82	0.775	0.280–2.146	0.830
	No	292	0.884	0.464–1.683	
Prior anticoagulant use	Yes	10	0.788	0.457–1.359	0.975
	No	364	N/A	N/A	
Initial NIHSS score	≥10	191	0.669	0.210–2.131	0.764
	>10	183	0.819	0.429–1.562	
Ischemic stroke types	LAA	87	1.219	0.410–3.623	0.061
	SVO	54	1.000	N/A	
	CE	118	**0.417**	**0.177–0.980**	
	Others	115	3.101	0.921–10.44	

*CE, cardioembolism; LAA, large-artery atherosclerosis; NIHSS, national institute of health stroke scale; SVO, small vessel occlusion*.

*N/A, not accessible because of the absence of or insufficient data on number of events*.

*Values in bold indicate statistical significance*.

## Discussion

The present study demonstrated that in the time window of 3–4.5 h after AIS onset, patients who received thrombolysis with intravenous alteplase exhibited functional improvement compared with controls, and the results were consistent across patient subgroups. Moreover, standard-dose and low-dose alteplase exhibited comparable effectiveness and safety in this time window, although the standard dose may be preferred in patients with atrial fibrillation or hypercholesterolemia.

The main novelty of our study is the comparison of the effectiveness of standard-dose and low-dose alteplase in the late time window, which has never been specifically investigated before. Japan proposed the use of low-dose alteplase at 0.6 mg/kg in 2006 on the basis of the results of an uncontrolled, open-label parallel study, in which the thrombolytic agent was administered within 3 h (16). This low-dose approach was soon adopted by neighboring Asian countries such as China, Korea, and Taiwan; however, several observational studies have reported contrary results regarding the benefit of the low-dose regimen (14, 17, 18). Notably, comparative studies using standard- or low-dose alteplase unquestionably extended the time limit to 4.5 h following the publication of the ECASS III trial in 2008, although the ECASS III trial only demonstrated the benefit of 0.9 mg/kg alteplase within 3–4.5 h (1).

To date, the strongest evidence of the effectiveness of low-dose alteplase administration within a window of 3–4.5 h was provided by the ENCHANTED trial published in 2016, which included patients who were given alteplase within 4.5 h of stroke onset (10). In the subgroup analysis of the ENCHANTED trial, no significant interaction was observed between the alteplase dose and time from onset to randomization (<3 vs. ≥3 h). In the ≥3-h subgroup (i.e., 3–4.5 h), the proportion of death and disability was 51.1% in the low-dose group and 50.1% in the standard-dose group (OR = 1.04, 95% CI = 0.84–1.30). In a study published in 2019 that included data of 6,250 patients from nine stroke registries in six Asian countries (the largest real-world data–based study to date) and compared the effectiveness of low-dose and standard-dose alteplase (11), the adjusted odds of death and disability or sICH were not significantly different between the low-dose and standard-dose groups. Although no significant interaction was observed between the alteplase dose and time (<3 vs. ≥3 h; *P*_*interaction*_ = 0.395), a trend favoring low-dose alteplase was noted in the ≥3-h subgroup (OR = 0.75 for death and disability, 95% CI = 0.51–1.10) compared with the <3-h subgroup (OR = 1.13, 95% CI = 0.93–1.37).

In comparison, our study only enrolled patients treated within a window of 3–4.5 h; this study might be the first matched-cohort study in Asia with a quasi-randomized control design to specifically consider this indication. The original goal of this study was to demonstrate the benefit of alteplase administered within a window of 3–4.5 h in comparison with the control. Further subgroup analyses revealed that the effectiveness and safety outcome were numerically higher in patients who were given standard-dose alteplase than in those who were given low-dose alteplase. Because of the observational study design, confounding by indication is inevitable, and physicians tend to prescribe low-dose alteplase for older patients. This is partly attributed to a finding reported by a multicenter study in Taiwan that a low dose of 0.6 mg/kg is associated with improved functional outcomes in elderly patients [71–80 years; (14)]. In our study, the overall effectiveness diminished with increasing age, irrespective of the alteplase dose used. Thus, more favorable outcomes may be observed in the standard-dose group. To overcome this selection bias, an age- and severity-matched cohort was created, and the results revealed a slightly greater number of favorable outcomes in the low-dose group than in the standard-dose group (36.9 vs. 33.9%). However, the aforementioned results were nonsignificant. Nevertheless, the present results do not preclude the use of a standard dose in older patients within the 3–4.5-h time window, even in those of Asian ethnicity.

A low-dose regimen, however, was associated with significantly lower sICH occurrence rate (1.0 vs. 2.1%, *P* = 0.01) in the ENCHANTED trial (10). Furthermore, a multicenter study in Taiwan revealed that in elderly patients (7180 years), the rate of sICH occurrence increased significantly as the dose increased (14). Our study, however, found that the rates of any ICH event and sICH occurrence were nonsignificantly higher in the low-dose group than in the standard-dose group. This may be related to the higher age in the low-dose group. However, no obvious interaction effect between age and the alteplase dose on ICH was observed (Figure 1), and the result remained consistent in age-adjusted or age-matched analyses. Certain unobserved factors in the low-dose group may have contributed to a higher sICH rate, or it could have occurred by chance given the low event rates. Our study results indicate that standard-dose alteplase within 3–4.5 h of stroke onset can be administered without causing an absolute increase in the risk of hemorrhage.

In the ECASS III trial, no heterogeneity in the treatment effect within a time window of 3–4.5 h was observed across all patient subgroups (19). Our study demonstrated similar benefits in various patient groups, including those groups that were excluded from the ECASS III trials, such as elderly patients (age > 80 years), those with concomitant diabetes mellitus and prior stroke, or those using anticoagulants. These findings are consistent with the 2019 American Stroke Association guideline for the early management of AIS, which stated that “careful analysis of available published data…indicates that these exclusion criteria from the trial may not be justified in practice” (8).

In the ENCHANTED trial, low-dose alteplase may have exerted a net benefit in patients without atrial fibrillation (20). Consistent with this finding, our study showed that patients with atrial fibrillation or hypercholesterolemia benefited more from standard-dose alteplase than from the low-dose option. In the histopathological composition of thrombi, atrial fibrillation–related cardioembolism is typically characterized by a higher percentage of fibrin with smaller fractions of red blood cells compared with noncardioembolic thrombi (21). Because alteplase specifically binds to fibrin and initiates fibrinolysis, patients with fibrin-rich thrombi may respond better to standard-dose than to low-dose alteplase. To rule out the age effect, age-adjusted or matched analyses were performed in the current study, which yielded findings similar to those of the aforementioned studies. The association between standard-dose alteplase and hypercholesterolemia is implicit and may also involve alteplase “resistance” in platelet-rich thrombi (22). Nevertheless, further studies are warranted to verify these results.

This is the first study to test the dose effect of alteplase given within the 3–4.5-h time window; this approach differs from that in subgroup analyses in previous larger studies (10, 11). Furthermore, we conducted sufficient subgroup evaluation to assess the heterogeneity of treatment effects. However, several limitations must be addressed. A major drawback of this study is the nonrandomized study design, due to which an imbalance in baseline characteristics may be observed between the treatment and control groups. However, we enrolled age- and sex-matched controls from the same hospital to minimize selection bias. Moreover, we adopted several statistical models to adjust the baseline imbalance. Second, the alteplase dose regimen was determined at the discretion of the physicians in charge, and this decision may be influenced by various clinical practices. Third, the sample size was relatively small and might limit the statistical power for detecting clinically meaningful differences. Fourth, the median door-to-needle time in our study was 65 min, which was longer than that in most clinical trials (23). However, our study started in 2008, at which time many hospitals in Taiwan had not implemented a dedicated code stroke yet (24). Physicians may also need to explain the off-label use of alteplase in the 3–4.5-h time window. These factors may significantly prolong the door-to-needle time. Nonetheless, our time metrics are comparable with the result of 65 min recorded in the participating hospitals of Get with the Guidelines—Stroke in the United States from 2006 to 2016 (25). Finally, patients treated with mechanical thrombectomy were excluded; thus, the results may not be extrapolated to patients with large vessel occlusion undergoing bridging reperfusion therapy. We did not collect information on the presence of intracranial large vessel occlusions. Therefore, we could not explore whether an interaction existed between the presence of large vessel occlusion and the efficacy of the standard-dose or low-dose alteplase, thus limiting the generalizability of our findings.

In summary, the present study demonstrated that the effectiveness of standard-dose alteplase may be comparable to that of low-dose alteplase in patients with AIS within a 3–4.5-h time window, without increasing the risk of hemorrhage. Additionally, standard-dose alteplase may be selected for patients with atrial fibrillation or hypercholesterolemia. In countries where the standard and low doses of alteplase are used in parallel, the current analysis may guide physicians in the selection of appropriate regimens.

## Data Availability Statement

The raw data supporting the conclusions of this article are available upon reasonable request to the corresponding author.

## Ethics Statement

The study was approved by the Institutional Review Board of National Taiwan University Hospital and informed consent was waived.

## Author Contributions

J-SJ contributed to the conception and design of the study. C-HaC and J-SJ analyzed the data. C-HaC contributed to the first draft of the manuscript. S-CT and J-SJ provided critical revision on the manuscript. All authors contributed to the acquisition of data and agreed with the final version of the manuscript.

## Conflict of Interest

The authors declare that the research was conducted in the absence of any commercial or financial relationships that could be construed as a potential conflict of interest.

## Publisher's Note

All claims expressed in this article are solely those of the authors and do not necessarily represent those of their affiliated organizations, or those of the publisher, the editors and the reviewers. Any product that may be evaluated in this article, or claim that may be made by its manufacturer, is not guaranteed or endorsed by the publisher.
