# Supplementary material for: Effectiveness of Standard-Dose vs. Low-Dose Alteplase for Acute Ischemic Stroke Within 3–4.5 h
Source: Front Neurol. 2022 Feb 8;13:763963. doi: 10.3389/fneur.2022.763963 (PMC8883875; doi:10.3389/fneur.2022.763963)
Supplement: Supplementary file 1 [file Table_1.DOCX]

**Effectiveness of Standard-dose versus Low-dose Alteplase for Acute Ischemic Stroke Within 3–4.5 Hours**

Supplemental Table 1– 3

**Supplemental Table 1.** Baseline characteristics between patients treated with alteplase and control.

| **Characteristics** | **Alteplase (n=374)** | **Control (n=374)** | ***P* value** |
| --- | --- | --- | --- |
| Age (year) | 66.1 ± 13.2 | 67.8 ± 12.5 | 0.290 |
| Age≧70 y, n (%) | 160 (42.8) | 183 (48.9) | 0.092 |
| Male, n (%) | 251 (67.1) | 251 (67.1) | 1.000 |
| Body weight (Kg) | 65.1 ± 12.7 | 65.4 ± 12.9 | **0.669** |
| Stroke subtype |  |  |  |
| LAA | 87 (23.3) | 97 (25.9) | 0.089 |
| SVO | 54 (14.4) | 75 (20.1) |  |
| CE | 118 (31.6) | 108 (28.9) |  |
| Others | 115 (30.8) | 94 (25.1) |  |
| NIHSS | 10 (7-17) | 9 (5-15) | **0.001** |
| Systolic BP (mmHg) | 161.0 ± 30.7 | 161.0 ± 32.5 | 0.434 |
| Diastolic BP (mmHg) | 91.6 ± 19.3 | 90.7 ± 19.2 | 0.680 |
| **Laboratory data** |  |  |  |
| Glucose (mg/dL) | 154.1 ± 69.8 | 158.5 ± 75.4 | 0.100 |
| INR | 1.01 ± 0.10 | 1.01 ± 0.10 | 0.987 |
| Creatinine (mg/dL) | 1.26 ± 1.09 | 1.35 ± 1.36 | 0.090 |
| Platelet count (10^5^/mm^3^) | 216.8 ± 72.0 | 212.8 ± 70.1 | 0.830 |
| **Medical history** |  |  |  |
| Hypertension | 282 (75.4) | 291 (77.8) | 0.490 |
| Diabetes mellitus | 137 (36.6) | 160 (42.8) | 0.100 |
| Previous stroke | 75 (20.1) | 94 (25.1) | 0.115 |
| Diabetes mellitus with previous stroke |  |  |  |
| Ischemic heart disease | 45 (12.0) | 38 (10.2) | 0.485 |
| Atrial fibrillation | 127 (34.0) | 113 (30.2) | 0.309 |
| Hyperlipidemia | 199 (53.2) | 201 (53.7) | 0.942 |
| Hypercholesterolemia | 174 (46.5) | 175 (46.8) | 1.000 |
| Hypertriglyceridemia | 67 (17.9) | 64 (17.1) | 0.847 |
| Ever smoking | 138 (34.2) | 137 (36.6) | 0.491 |
| Current smoker | 103 (27.5) | 98 (26.2) | 0.680 |
| Prior antiplatelet use |  |  |  |
| Prior anticoagulant use | 5 (2.8 |  |  |
| **Outcome** |  |  |  |
| mRS 0-1 | 127 (34.0) | 85 (22.7) | **0.001** |
| mRS 0-2 | 179 (47.9) | 150 (40.1) | **0.033** |
| END | 56 (15.0) | 73 (19.5) | 0.100 |
| Any ICH | 65 (17.4) | 32 (8.6) | **0.0003** |
| Symptomatic ICH | 13 (3.5) | 9 (2.4) | 0.387 |

Abbreviation: BP, blood pressure; CE, cardioembolism; END, early neurological deterioration; ICH, intracerebral hemorrhage; INR, international normalized ratio; mRS, modified Rankin Scale; LAA, large-artery atherosclerosis; NIHSS, National Institute of Health Stroke Scale; SVO, small vessel occlusion.

**Supplemental Table 2.** Univariate analysis of variables associated with clinical outcome.

| **Characteristics** | **mRS 0-1** | **END** | **Any ICH** |
| --- | --- | --- | --- |
| Age (year) | **0.96 (0.94-0.98)** | 1.00 (0.98-1.02) | 1.02 (0.99-1.04) |
| Male, n (%) | 1.16 (0.73-1.84) | 0.79 (0.44-1.42) | **0.47 (0.27-0.80)** |
| NIHSS | **0.87 (0.83-0.90)** | **1.05 (1.01-1.09)** | **1.13 (1.08-1.17)** |
| Systolic BP (mmHg) | 1.00 (0.99-1.01) | 1.01 (0.99-1.01) | 1.01 (0.99-1.01) |
| Diastolic BP (mmHg) | 1.00 (0.99-1.01) | 1.01 (0.99-1.02) | 1.01 (0.99-1.02) |
| Glucose (mg/dL) | **0.996 (0.992-0.999)** | **1.01 (1.00-1.01)** | 1.002 (0.998-1.005) |
| INR | 0.10 (0.01-1.30) | 2.68 (0.18-39.9) | 5.63 (0.49-64.4) |
| Creatinine (mg/dL) | 0.96 (0.78-1.19) | 0.92 (0.67-1.27) | 1.03 (0.81-1.30) |
| Platelet count (10^5^/mm^3^) | 1.00 (0.997-1.003) | 1.00 (0.99-1.01) | 0.998 (0.994-1.002) |
| Hypertension | 0.66 (0.40-1.06) | 1.40 (0.69-2.83) | 1.37 (0.71-2.66) |
| Diabetes mellitus | **0.46 (0.28-0.73)** | **2.71 (1.52-4.82)** | **2.19 (1.28-3.77)** |
| Previous ischemic stroke | **0.27 (0.14-0.54)** | 0.53 (0.23-1.21) | 1.12 (0.58-2.15) |
| Ischemic heart disease | 0.77 (0.39-1.52) | 0.86 (0.35-2.13) | 1.42 (0.67-3.05) |
| Atrial fibrillation | **0.61 (0.38-0.97)** | **2.03 (1.14-3.61)** | **3.48 (2.00-6.04)** |
| Hyperlipidemia | 1.02 (0.67-1.57) | 1.31 (0.74-2.34) | 0.77 (0.45-1.31) |
| Hypercholesterolemia | 0.95 (0.62-1.46) | 1.52 (0.86-2.69) | 0.78 (0.46-1.35) |
| Hypertriglyceridemia | 0.87 (0.49-1.53) | 1.48 (0.75-2.94) | 0.80 (0.39-1.67) |
| Ever smoking | 0.98 (0.62-1.53) | 1.18 (0.66-2.13) | 0.57 (0.31-1.06) |
| Current smoker | 1.27 (0.79-2.03) | 1.30 (0.70-2.40) | 0.61 (0.32-1.17) |

Abbreviation: BP, blood pressure; INR, international normalized ratio; NIHSS, National Institute of Health Stroke Scale.

**Supplemental Table 3.** Baseline characteristics between matched patients with standard or low dose.

| **Characteristics** | **Standard dose (n=65)** | **Low dose (n=65)** | ***P* value** |
| --- | --- | --- | --- |
| Age (year) | 68 (59-74) | 68 (59-74) | 1.000 |
| Age≧70 y, n (%) |  |  |  |
| Male, n (%) | 40 (61.5) | 43 (66.2) | 0.584 |
| Body weight (Kg) | 60 (55-65) | 66 (62-70) | **0.001** |
| Stroke subtype |  |  |  |
| LAA | 15 (23.1) | 19 (29.2) | 0.872 |
| SVO | 12 (18.5) | 10 (15.4) |  |
| CE | 20 (30.8) | 19 (29.2) |  |
| Others | 18 (27.7) | 17 (26.2) |  |
| NIHSS | 10 (7-15) | 10 (7-15) | 0.909 |
| Systolic BP (mmHg) | 165 (141-190) | 158 (144-183) | 0.625 |
| Diastolic BP (mmHg) | 95 (81-110) | 90 (80-101) | 0.161 |
| **Laboratory data** |  |  |  |
| Glucose (mg/dL) | 129 (107-166) | 121 (104-166) | 0.812 |
| INR | 0.99 (0.95-1.07) | 0.96 (0.93-1.03) | 0.083 |
| Creatinine (mg/dL) | 1.01 (0.85-1.50) | 0.96 (0.78-1.30) | 0.186 |
| Platelet count (10^5^/mm^3^) | 221 (175-267) | 212 (181-240) | 0.610 |
| **Medical history** |  |  |  |
| Hypertension | 52 (80.0) | 52 (80.0) | 1.000 |
| Diabetes mellitus | 22 (33.9) | 23 (35.4) | 0.854 |
| Previous stroke | 14 (21.5) | 13 (20.0) | 0.829 |
| Diabetes mellitus with previous stroke | 7 (10.8) | 7 (10.8) | 1.000 |
| Ischemic heart disease | 5 (7.7) | 9 (13.9) | 0.258 |
| Atrial fibrillation | 21 (32.3) | 20 (30.8) | 0.850 |
| Hyperlipidemia | 42 (64.6) | 39 (60.0) | 0.587 |
| Hypercholesterolemia | 31 (47.7) | 35 (53.9) | 0.483 |
| Hypertriglyceridemia | 19 (29.3) | 13 (20.0) | 0.222 |
| Ever smoking | 16 (24.6) | 28 (43.1) | **0.026** |
| Current smoker | 15 (23.1) | 20 (30.8) | 0.323 |
| Prior antiplatelet use | 16 (24.6) | 20 (30.8) | 0.433 |
| Prior anticoagulant use | 1 (1.5) | 2 (3.1) | 1.000 |
| **Outcome** |  |  |  |
| mRS 0-1 | 22 (33.9) | 24 (36.9) | 0.714 |
| mRS 0-2 | 32 (49.2) | 33 (50.8) | 0.861 |
| END | 10 (15.4) | 11 (16.9) | 0.812 |
| Any ICH | 9 (13.9) | 10 (15.4) | 0.804 |
| Symptomatic ICH | 1 (1.5) | 2 (3.1) | 1.000 |

Abbreviation: BP, blood pressure; CE, cardioembolism; END, early neurological deterioration; ICH, intracerebral hemorrhage; INR, international normalized ratio; mRS, modified Rankin Scale; LAA, large-artery atherosclerosis; NIHSS, National Institute of Health Stroke Scale; SVO, small vessel occlusion.
